# The guanine nucleotide exchange factor Ric-8A regulates the sensitivity of constitutively active Gαq to the inhibitor YM-254890

**DOI:** 10.1016/j.jbc.2025.108426

**Published:** 2025-03-19

**Authors:** Morgan B. Dwyer, Jiansong Luo, Tyson D. Todd, Kendall J. Blumer, Gregory G. Tall, Philip B. Wedegaertner

**Affiliations:** 1 Department of Biochemistry and Molecular Biology, Sidney Kimmel Medical College, Thomas Jefferson University, Philadelphia, Pennsylvania, USA; 2 Department of Cell Biology and Physiology, Washington University, Saint Louis, Missouri, USA; 3 Department of Pharmacology, University of Michigan Medical School, Ann Arbor, Michigan, USA

**Keywords:** cell signaling, GEF, GTPase, heterotrimeric G protein, oncogene, plasma membrane

## Abstract

Heterotrimeric G proteins are stimulated under normal circumstances by G protein–coupled receptors to promote downstream intracellular signaling. Mutations can occur in αq at glutamine 209 (Q209) that cause constitutive, G protein–coupled receptor independent signaling due to disruption of GTPase activity. Specifically, Q209L/P mutations are oncogenic drivers of uveal melanoma. YM-254890 (YM) has been shown to selectively inhibit both WT and constitutively active (CA) αqQ209L/P by preventing the release of GDP and exchange for GTP, thereby halting downstream signaling. Because αqQL/P are thought to be primarily GTP-bound and GTPase deficient, the current mechanistic understanding of YM inhibition needs further investigation to clarify how a GDP-dissociation inhibitor could potently inhibit these oncogenic mutants. Here, we expand on the current knowledge of CA αq cellular regulation by demonstrating a direct role for the αq chaperone and guanine nucleotide exchange factor Ric-8A in YM sensitivity. Through signaling assays in *RIC-8A* KO cells, we found that myristoylated αqQL/P mutants (αqAG-QL/P), previously demonstrated to be YM-resistant, became YM-sensitive, and this was reversed by reintroduction of Ric-8A. Additionally, αqQL demonstrated increased YM sensitivity in the absence of Ric-8A, which was directly altered by the reintroduction of Ric-8A. Pull-down and BRET assays with the RGS-homology domain of GRK2, which can only bind activated αq, further demonstrated that Ric-8A expression enhances activation of αq, its ability to bind effectors, and therefore its ability to signal. With the understanding of YM acting as a GDP-dissociation inhibitor, we propose that Ric-8A hinders YM inhibitory effects by promoting GTP-bound, activated αqQL/P.

Heterotrimeric G proteins are canonically regulated by ligand-induced G protein–coupled receptor (GPCR) activation. These activated receptors act as guanine nucleotide exchange factors (GEFs) for the α subunit by promoting GDP/GTP exchange, leading to dissociation of the heterotrimer. Gα-GTP and the Gβγ dimer then interact with their downstream targets (1, 2, 3, 4, 5). Gα subunits have intrinsic GTPase activity that can be enhanced by GTPase-activating proteins (GAPs) of the regulator of G protein signaling (RGS) family, which halts signaling and leads to subsequent reformation of the heterotrimer of Gβγ with Gα-GDP (6, 7). There are four families of Gα, consisting of αs, αi, αq, and α12/13, that have different roles in various cellular signaling cascades (8). These studies will focus on αq, which belongs to the same family as α11, α14, and α15/16. Canonically, these GPCR-activated αq proteins stimulate phospholipase Cβ, (PLCβ), which hydrolyzes phosphatidylinositol 4,5-bisphosphate to produce second messengers inositol 1,4,5-trisphosphate (IP3) and diacylglycerol (DAG) (9, 10, 11). DAG activates protein kinase C (PKC) while inositol 1,4,5-trisphosphate regulates calcium release from the endoplasmic reticulum into the cytoplasm. αq can also directly activate RhoGEFs p63, Trio, and kalirin (12).

In some cases, Gα can be mutationally activated and act independently of receptor activation. Constitutively activating driver mutations have been found in the subunits αq and α11 in uveal melanoma, the most common intraocular tumor in adults, with metastasis occurring primarily to the liver in up to 50% of patients. Metastasis is often undetectable and unavoidable even when the primary tumor is treated (13). αq and α11, which share 90% identity, mutually exclusively contain mutations at glutamine (Q) 209 and less commonly arginine (R) 183 in uveal melanoma. Q209 lies in the critical switch II region in the GTPase domain of αq and is essential for intrinsic GTP hydrolytic activity. This residue helps stabilize the γ phosphate of GTP and the transition state for GTP hydrolysis. When Q209 is mutated, almost exclusively to leucine or proline in uveal melanoma, GTP-bound αq becomes constitutively active (CA) due to an inability to hydrolyze GTP (14).

In uveal melanoma, mutant αqQ209L (αqQL) and αqQ209P (αqQP) promote aberrant cellular signaling that drives oncogenesis (15, 16, 17, 18). Specifically, the mitogen-activated kinase (MAPK) cascade and the activation and nuclear translocation of yes-associated protein (YAP) are two major pathways activated by CA αqQL. Downstream of PLCβ activation through CA αq, DAG recruits PKC and Ras exchange factor RasGRP3 to the membrane where RasGRP3 is phosphorylated by PKC, leading to an increase in activated Ras that then stimulates the Raf/Mek/Erk MAPK cascade (19, 20, 21). YAP, a transcriptional coactivator for TEAD transcription factors and mediator of the Hippo pathway, is activated by αq interaction with Trio RhoGEF, which activates RhoA. Ultimately RhoA releases YAP from cytosolic sequestering proteins by increasing polymerized actin that displaces YAP in binding these proteins. Free YAP can then translocate to the nucleus to promote transcription (22, 23). Another critical component in the activation of YAP is focal adhesion kinase, also activated downstream of Trio/RhoA. Focal adhesion kinase negatively regulates MOB1 of the Hippo pathway through phosphorylation, while also activating and stabilizing free YAP. Therefore, coordination between the inactivation of the Hippo pathway with actin-mediated accumulation of free YAP leads to high levels of activated YAP, that in a cancer context, promotes tumorigenesis and proliferation (24, 25, 26).

YM-254890 (YM), isolated from Chromobacterium, and FR900359 (FR), isolated from *Ardisia crenata*, are both cyclic depsipeptides of highly similar structure that selectively inhibit both WT and CA αq (27, 28, 29, 30, 31). Structural and biochemical studies have shown that both inhibit αq/11/14 by acting as guanine nucleotide dissociation inhibitors to prevent GDP release and keep them locked in an inactive conformation (30, 32). However, a paradox exists regarding how a presumably constitutively GTP-bound αq would be susceptible to the inhibitory effects of YM/FR. It is not currently known how αqQL would be sufficiently in a GDP-bound form necessary for inhibition (33). Though the mechanism is not fully clear, YM/FR make great tools for better understanding the biochemistry of these CA αq and their vulnerabilities to better grasp how to attenuate their signaling.

Previous work in our lab shed light on the question of how YM could inhibit signaling by CA αq by proposing a novel mechanism whereby YM promotes a subcellular redistribution of αqQL off of the plasma membrane (PM) (34). In those studies, we took advantage of an engineered N terminally myristoylated mutant, called αqAG-QL, in which residues 2 to 7 were deleted and alanine 8 was mutated to a glycine to introduce a new N-terminal myristoylation site. Together, this new myristoylation and dual palmitoylation of cysteines 9 and 10 enhanced membrane binding and allowed αqAG-QL to remain at the PM in the presence of YM. Signaling by this PM-restricted αqAG-QL was not inhibited by YM, consistent with the idea that YM may inhibit CA αqQL by promoting its release from the PM (34). Yet, how αqAG-QL evades inhibition by YM and what cellular factors might influence the ability of αqQL to be inhibited by YM are not understood.

In this report, we provide further insight into these questions. First, we show that addition of an N-terminal myristoylation site to enhance membrane binding of αqQP and a YM-sensitive mutant of α13QL leads to loss of YM inhibition of signaling by these CA Gα. Second, we show that loss of Ric-8A, a chaperone and GEF for Gα, directly impacts YM sensitivity of CA αq. Ric-8A binds to and stabilizes newly synthesized, nucleotide-free Gα and promotes GTP binding to Gα, which is then released from the chaperone. Beyond initiating the first GTP-binding event on nascent αq in cells, it is not clear that Ric-8A has a role in further activating WT or CA αq (35, 36, 37, 38). Here, we discovered that loss of Ric-8A allows αqAG-QL to gain the ability to be inhibited by YM, while reexpression of exogenous Ric-8A returns αqAG-QL to being resistant to YM inhibition. Moreover, we show that Ric-8A can regulate the sensitivity of αqQL to YM, providing the first identification of a protein that regulates the ability of CA αq to be inhibited by YM.

## Results

### YM-resistance conferred by myristoylation extends beyond **α**qQL

**Figure 1:**
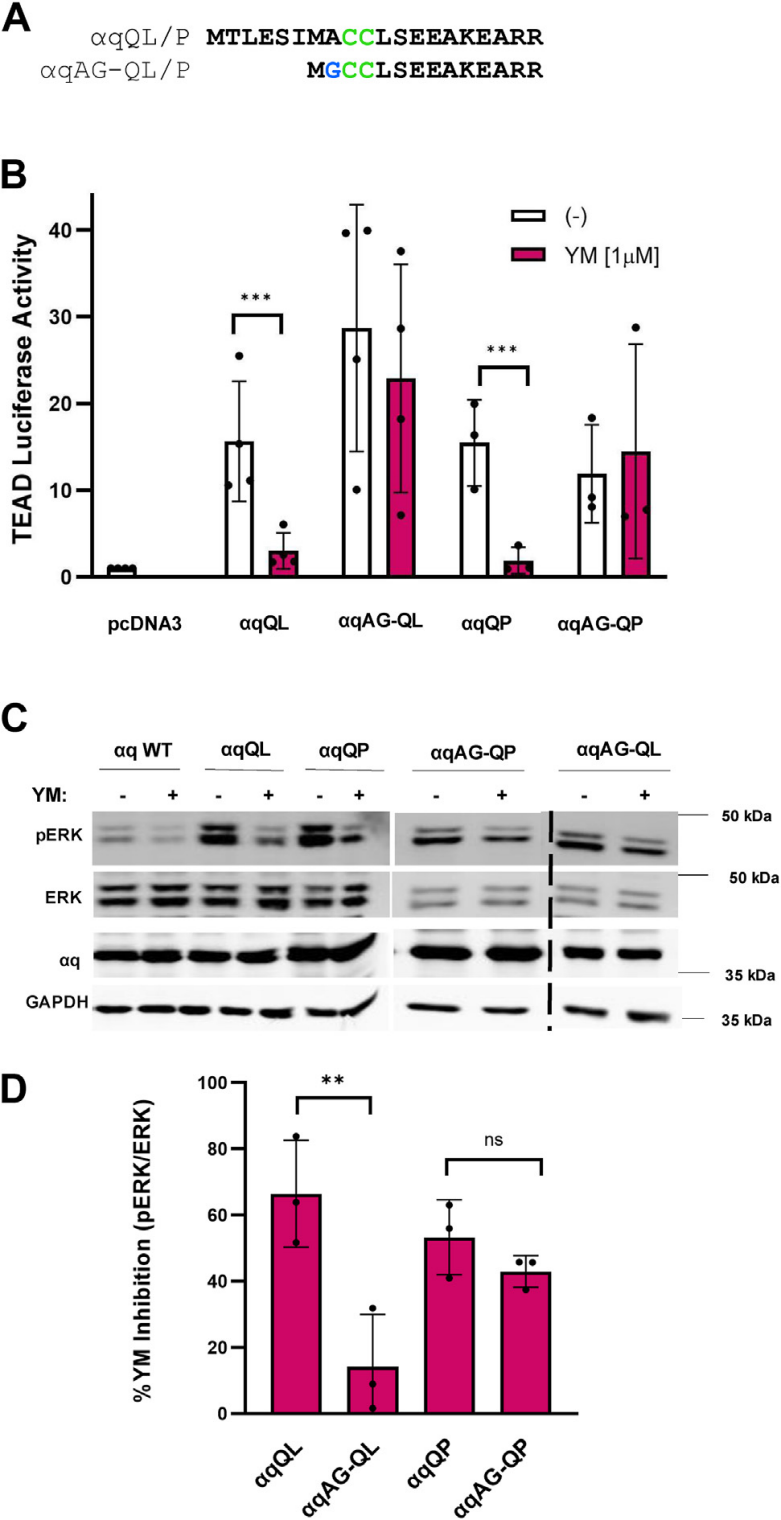
**Myristoylation of αqQL and αqQP confer YM-resistance.***A*, sequence diagram of the first 19 residues of αqQL/P depicting the changes made to generate a myristoylation sequence. *Green* colored cysteine residues (*C*) correspond to palmitoylation sites present in both myristoylated and non-myristoylated αqQL/P. Alanine (*A*) is mutated to the *blue* colored glycine (G) at position 2 in αqAG-QL/P, which introduces the myristoylation site. *B*, TEAD luciferase 8X-GTIIC reporter and renilla luciferase were cotransfected into HEK293 q/11 KO cells along with either pcDNA3 (negative control), αqQL/P, or αqAG-QL/P. Two hours after transfection, media were changed to serum-free and 1 μM YM was added overnight where indicated. Cells were lysed the following day, and luciferase experiments were performed. TEAD luciferase values were normalized to renilla luciferase values for the same well and averaged for each condition. Values are plotted as fold change over DNA. *C*, representative pERK immunoblotting in HEK293 q/11 KO cells. Cells were transfected with either pcDNA3 (negative control), αqQL/P, or αqAG-QL/P and 24 h later media were changed to serum-free and 1 μM YM was added where indicated. The next day cell, lysates were collected and immunoblotted for pERK, ERK, αq, and GAPDH. *D*, quantification of signal intensities from three independent pERK experiments. Phosphorylated ERK was divided by total ERK. Values are shown as percentage YM inhibition compared to the respective nontreated, basal pERK signal. Results are shown as mean ± SD and statistical significance is indicated. (n = 3 or 4, ∗∗*p* < 0.01; ∗∗∗*p* < 0.005, two-way ANOVA, Šidák's multiple comparison’s test). pERK, phosphorylated ERK.

**Figure 2:**
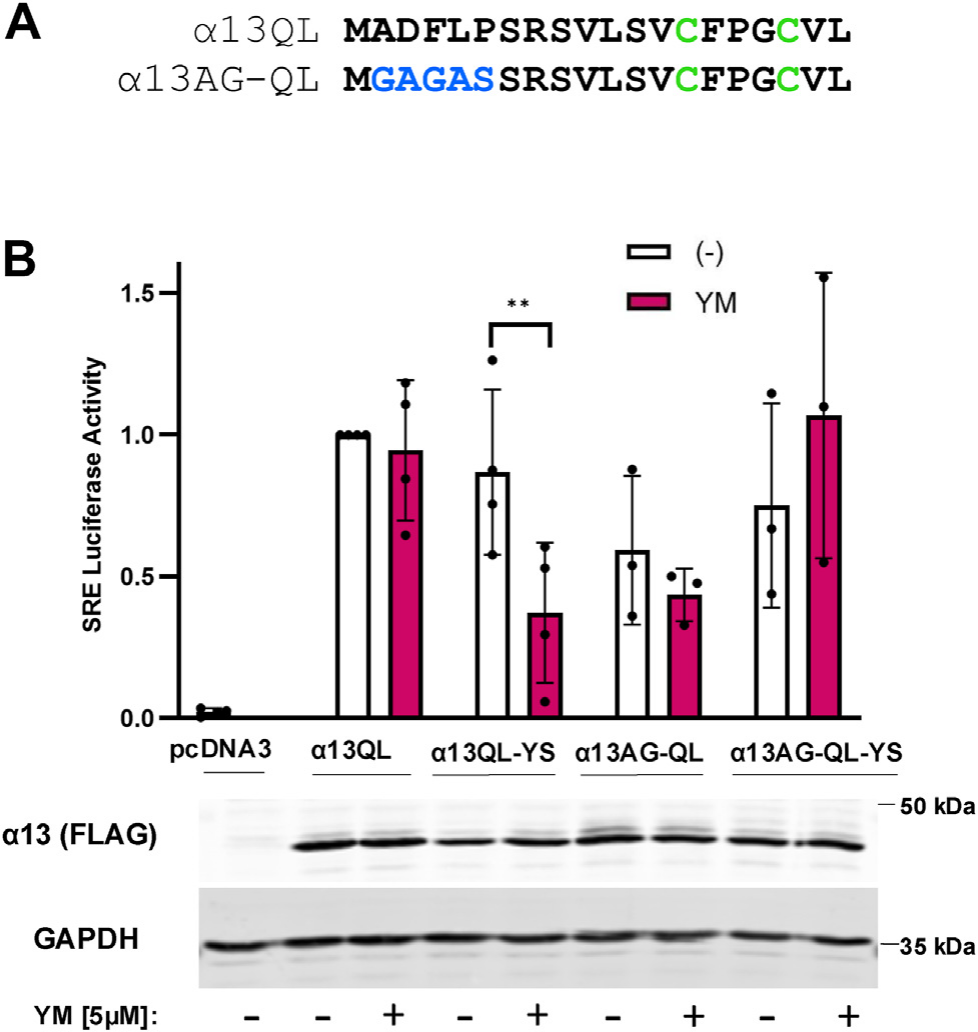
**Myristoylation prevents YM inhibition of YM-sensitive CA α13QL.***A*, sequence diagram depicting the N-terminal changes made to α13QL to introduce a myristoylation sequence. *Blue* colored residues represent the introduced myristoylation site and the *green* colored residues represent the palmitoylated cysteines that are present in both α13QL and in α13AG-QL. *B*, SRE-luciferase reporter assay performed in HEK293 cells transfected with pcDNA3, α13QL, α13QL-YS (YM-sensitive), α13AG-QL, and α13AG-QL-YS. Cells were cotransfected with SRE and renilla luciferase reporters. Two hours after transfection, media were changed to serum-free and 5 μM YM was added overnight. The next day cells were lysed and luciferase assays were run. SRE luciferase values were normalized to renilla luciferase values for each well and averaged for each condition. Results are shown as mean ± SD, normalized to α13QL as 1, and statistical significance is indicated. (n = 3 or 4, ∗∗*p* < 0.01, two-way ANOVA, Šidák's multiple comparison’s test). The α13 constructs were FLAG-tagged, and an anti-FLAG antibody was used to detect expression of the α13 proteins (*bottom*) and the order of samples corresponds to the above graph. SRE, serum response element.

As described above, our previous work demonstrated that αqAG-QL, an αqQL mutant engineered to undergo myristoylation and thus enhanced PM binding, was resistant to inhibition by YM (34). Here, in this manuscript, we asked if this YM resistance could be extended to an additional activating mutation in αq and to an additional Gα subunit (Figs. 1 and 2) (34). Other than Q209L, Q209P is the most widely reported mutant in uveal melanoma. Thus, we introduced N-terminal myristoylation into CA αq-QP, to create αqAG-QP. This mutant was generated exactly as described previously for αqAG-QL (34) (Fig. 1*A*). We assayed the impact of YM treatment on signaling by αqQP and αqAG-QP with a TEAD luciferase reporter that measures activation of the YAP pathway. HEK293 cells with CRISPR deletions of αq and α11 (HEK293 q/11 KO) were used for these assays. As we described previously, αqQL is fully sensitive to YM inhibition of signaling while myristoylated αqAG-QL is resistant (Fig. 1*B*) (34). Likewise, we now show that αqQP is completely inhibited by YM, but αqAG-QP is not inhibited by YM (Fig. 1*B*). We also used immunoblotting for phosphorylated extracellular signal-regulated kinase (pERK) levels as another way to assess the constitutive activity of αq mutants. Similarly to our luciferase reporter data, αqQL and αqQP constitutive stimulation of pERK was strongly inhibited by treatment with YM (Fig. 1, *C* and *D*). αqAG-QL was significantly less sensitive to YM than αqQL, as demonstrated previously (34). αqAG-QP YM-sensitivity was slightly and consistently decreased, but this did not reach statistical significance (39, 40), highlighting that the proline and leucine mutations are not entirely identical. These results indicate that αqQP, similarly to αqQL, displays reduced sensitivity to the inhibitor YM upon introduction of a single myristoylation site.

To better understand if there is something specific about αqAG-QL that confers resistance to YM in addition to its myristoylation, we examined a Gα from a different family: α13. Are there other features, outside of N-terminal lipid modifications, that allow it to evade the effects of YM? A constitutively activated α13, containing a leucine substitution for the conserved glutamine 226, was engineered to create a YM-binding site by replacing multiple α13 residues with ones from αq (α13QL-YS), as previously described (41). In addition to the residues that were already changed to make it YM-sensitive, we introduced a myristoylation sequence by substituting the N-terminal six amino acids in α13QL and α13QL-YS with a myristoylation consensus sequence from transducin (Fig. 2*A*). Using a serum response element (SRE) transcriptional reporter assay in HEK293 cells, we found that overexpressed, CA α13QL stimulated SRE luciferase activity but was unaffected by YM, as expected. α13QL-YS signaled comparably to α13QL but was significantly inhibited by YM. Subsequently, 5 μM YM was used here based on our previous work demonstrating that α13QL-YS, previously termed α13qQL, was substantially less sensitive to FR compared to αqQL (41). Interestingly, myristoylated α13AG-QL-YS, like αqAG-QL, was not inhibited by YM and maintained strong SRE-luciferase signaling in the presence or absence of YM (Fig. 2*B*). These data suggest that aside from the constitutively activating glutamine to leucine/proline mutation and the myristoylation site, no other features specific to αq are required for the loss of sensitivity to YM. These experiments demonstrate that a single myristoylation site is sufficient for CA Gα to overcome the effects of YM and maintain signaling, and this is not limited to the αq background.

### Ric-8A expression affects YM-sensitivity of CA **α**qAG-QL and **α**qQL

**Figure 3:**
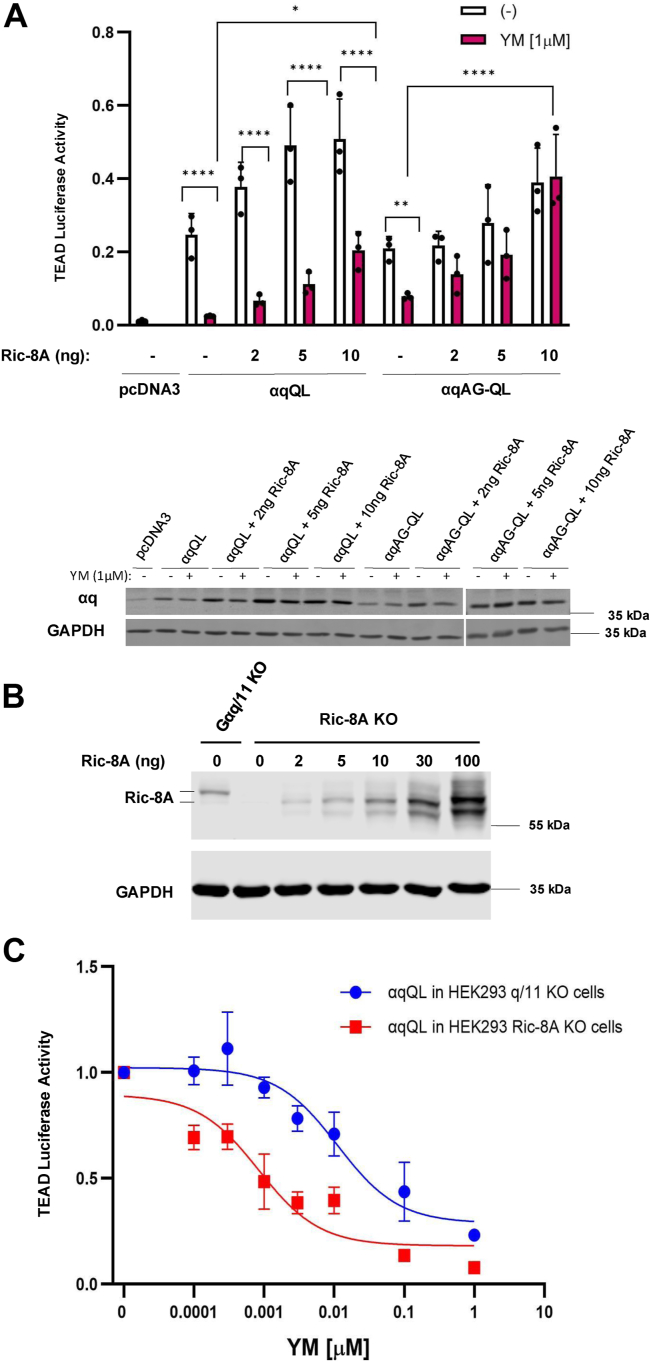
**Loss of Ric-8A leads to YM-sensitivity of αqAG-QL and increases sensitivity of αqQL to YM.***A*, TEAD luciferase assay in *RIC-8A* KO cells transfected with either pcDNA3 (negative control), αqQL, αqAG-QL, and increasing amounts of *RIC-8A* as indicated. TEAD luciferase 8X-GTIIC reporter and renilla luciferase were cotransfected. Two hours after transfection, media were changed to serum-free and 1 μM YM was added overnight where indicated. Cells were lysed the following day, and luciferase experiments were performed. TEAD luciferase values were normalized to renilla luciferase values for the same well and averaged for each condition. Results are shown as mean ± SD and statistical significance is indicated. (n = 3, ∗*p* < 0.05; ∗∗*p* < 0.01; ∗∗∗∗*p* < 0.0001, two-way ANOVA, Šidák's multiple comparison’s test). αq protein expression was assessed by immunoblotting. *B*, immunoblot for Ric-8A demonstrating endogenous levels in q/11 KO cells in comparison to exogenously expressed levels in *RIC-8A* KO cells. Endogenous Ric-8A migrates slightly slower than the plasmid-expressed Ric-8A, and this is indicated by the two lines at the Ric-8A immunoblot. This difference may be due to alternative methionine initiation or slight proteolysis of the exogenously expressed Ric-8A. *C*, YM IC50 analysis readout by the TEAD luciferase reporter comparing *RIC-8A* KO and q/11 KO cells transfected with αqQL and treated with YM concentrations ranging from 0.0001 to 1 μM. YM treatments were overnight in serum-free media (n = 3).

It is clear that enhanced membrane targeting plays a role in YM resistance, but why this confers resistance is unclear. We hypothesized that proteins that interact with αq could regulate the sensitivity to YM inhibition of signaling, and that such regulation may be particularly important for CA forms of αq. Ric-8A is an attractive candidate; it is a key regulator of αq, having chaperone and GEF activities (35, 36, 37, 38). To test the potential role of Ric-8A on the YM-resistance exhibited by αqAG-QL, we utilized HEK293 cells with a CRISPR/Cas9-mediated deletion of *RIC-8A* (*RIC-8A* KO cells). Surprisingly, αqAG-QL demonstrated significant sensitivity to YM in the TEAD luciferase reporter assay when expressed in *RIC-8A* KO cells (Fig. 3*A*). This marks the first cellular context in which we observed YM inhibition of αqAG-QL signaling. Notably, reintroducing low amounts of transfected *RIC-8A* leads to YM-resistance of αqAG-QL without dramatically altering basal signaling or expression of either αqQL or αqAG-QL (Fig. 3*A*). Both αqQL and αqAG-QL were still strongly detected in the total absence of Ric-8A, suggesting that the sensitivity changes we see are not products of the protein not being produced (Fig. 3*A*). In comparing Ric-8A levels in q/11 KO and *RIC-8A* KO cells, it is evident that the exogenous levels we used resemble endogenous expression of Ric-8A in q/11 KO cells (Fig. 3*B*). Interestingly, αqQL begins to demonstrate some loss of YM-sensitivity with increasing amounts of Ric-8A (Fig. 3*A*). Next, we used the TEAD luciferase assay to compare YM potency of inhibition of αqQL in *RIC-8A* KO cells to q/11 KO cells. Increasing concentrations of YM from 0.1 nM to 1 μM were used to determine inhibition of αqQL (Fig. 3*C*). Interestingly, the lack of Ric-8A leads to a shift in the YM IC_50_ for αqQL inhibition from 11.2 nM in q/11 KO cells to 0.8 nM in *RIC-8A* KO cells, suggesting a direct role for Ric-8A in YM-sensitivity of oncogenic αqQL (Fig. 3*C*). Therefore, by its GEF activity and/or chaperone function, Ric-8A permits evasion of YM-inhibition of αqAG-QL and of αqQL to a lesser extent. These results mark the first time the potency of YM for any αq protein was notably altered by the effects of another protein.

**Figure 4:**
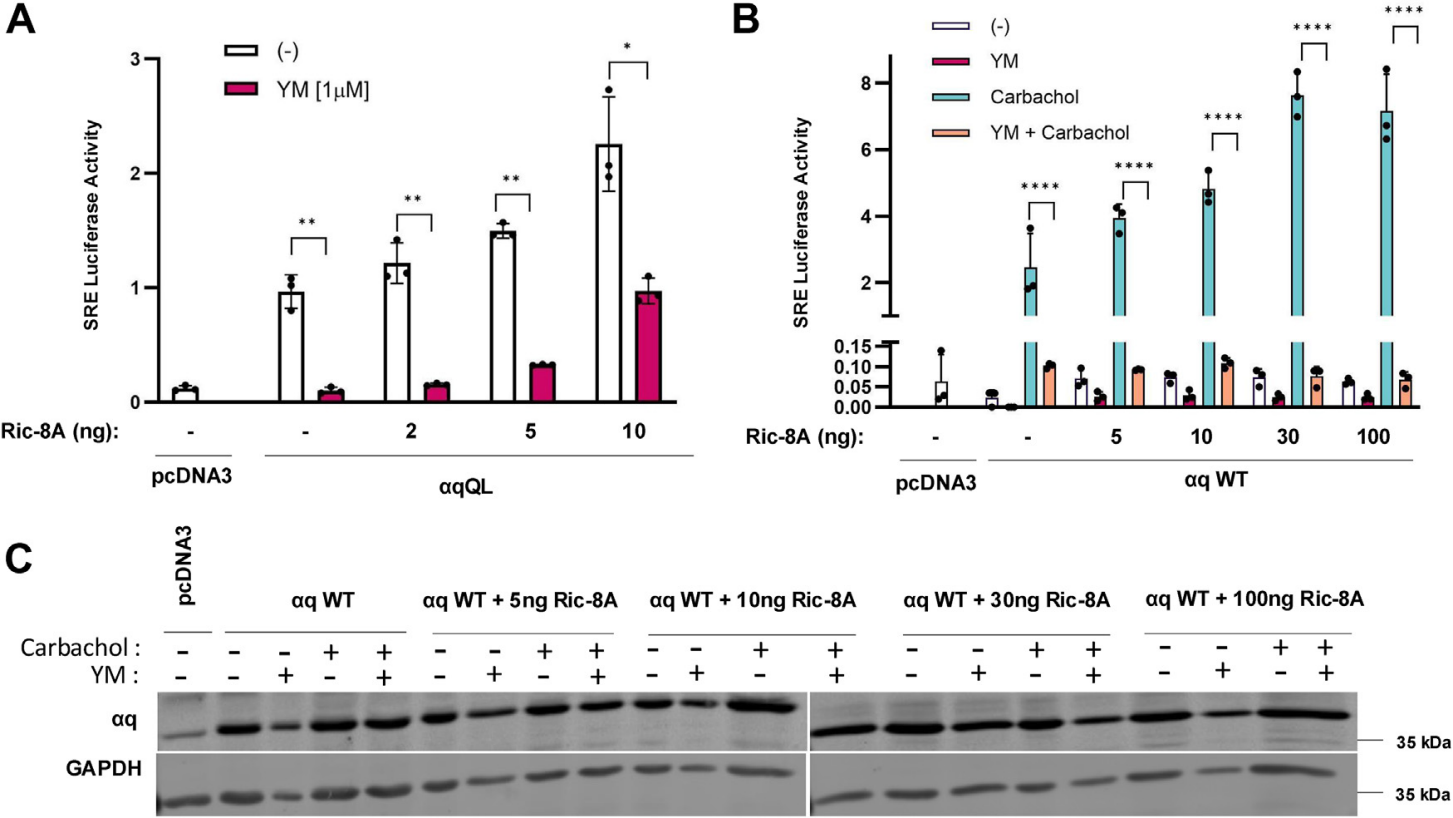
**Ric-8A only exerts its effects on YM-sensitivity in the context of CA αq, not αq WT.***A* and *B*, SRE luciferase reporter assays in *RIC-8A* KO cells transfected with pcDNA3 (negative control), αqQL, or αq WT, and increasing amounts of *RIC-8A* where indicated, along with SRE and renilla luciferase plasmids. For αq WT experiments (*B*), m3AChR was coexpressed. Two hours after transfection, media were changed to serum-free and 1 μM YM was added overnight where indicated. Cells were lysed the following day, and luciferase experiments were run. *B*, to stimulate αq WT, 100 μM carbachol was added 1 h after YM-treatment. SRE values are normalized to respective renilla values for the same well and averaged for each condition. Results are shown as mean ± SD and statistical significance is indicated. (n = 3, ∗*p* < 0.05; ∗∗*p* < 0.01; ∗∗∗∗*p* < 0.0001, two-way ANOVA, Šidák's multiple comparison’s test). *C*, lysates were immunoblotted to assess αq protein levels. m3AChR, muscarinic acetylcholine m3 receptor; SRE, serum response element.

The surprising finding that the αqQL response to YM was directly impacted by Ric-8A expression (Fig. 3) prompted us to test if αq WT behaves similarly. Both αq WT and αqQL are susceptible to YM, so the question is raised whether αq WT experiences a similar decline in YM-sensitivity with increasing amounts of Ric-8A produced in *RIC-8A* KO cells, or if this is a feature unique to GTPase-deficient αq, such as αqQL. To first validate our findings with αqQL, we performed SRE luciferase reporter assays, an orthogonal signaling assay previously characterized to be a robust readout of agonist-activated αq WT, as well as constitutive signaling by αqQL. Similar to the TEAD luciferase assays (Fig. 3*A*), SRE luciferase assays showed that αqQL signaling in *RIC-8A* KO cells was almost completely inhibited by YM, but, strikingly, αqQL gained partial resistance to YM when increasing amounts of Ric-8A were expressed, corroborating that Ric-8A directly plays a role in YM-resistance (Fig. 4*A*). Next, to examine GPCR-activated αq WT in this context, the muscarinic acetylcholine m3 receptor (m3AChR) was coexpressed with αq WT. Addition of the m3AChR agonist carbachol to activate signaling by αq WT resulted in robust stimulation of SRE luciferase, and this activation was inhibited by >90% by treatment with YM (Fig. 4*B*). However, in contrast to the effect of reexpressing Ric-8A on αqQL signaling (Fig. 4*A*), expression of increasing amounts of Ric-8A in the *RIC-8A* KO cells failed to result in any loss of YM inhibition of αq WT (Fig. 4*B*). Immunoblots for αq WT demonstrated that protein was produced at comparable levels under each condition (Fig. 4*C*). Together, these results show that αq WT retains potent inhibition by YM independent of Ric-8A levels, indicating that the ability of Ric-8A to regulate sensitivity to YM is specific to CA αqQL.

### Ric-8A directly impacts YM sensitivity of **α**qAG-QP

**Figure 5:**
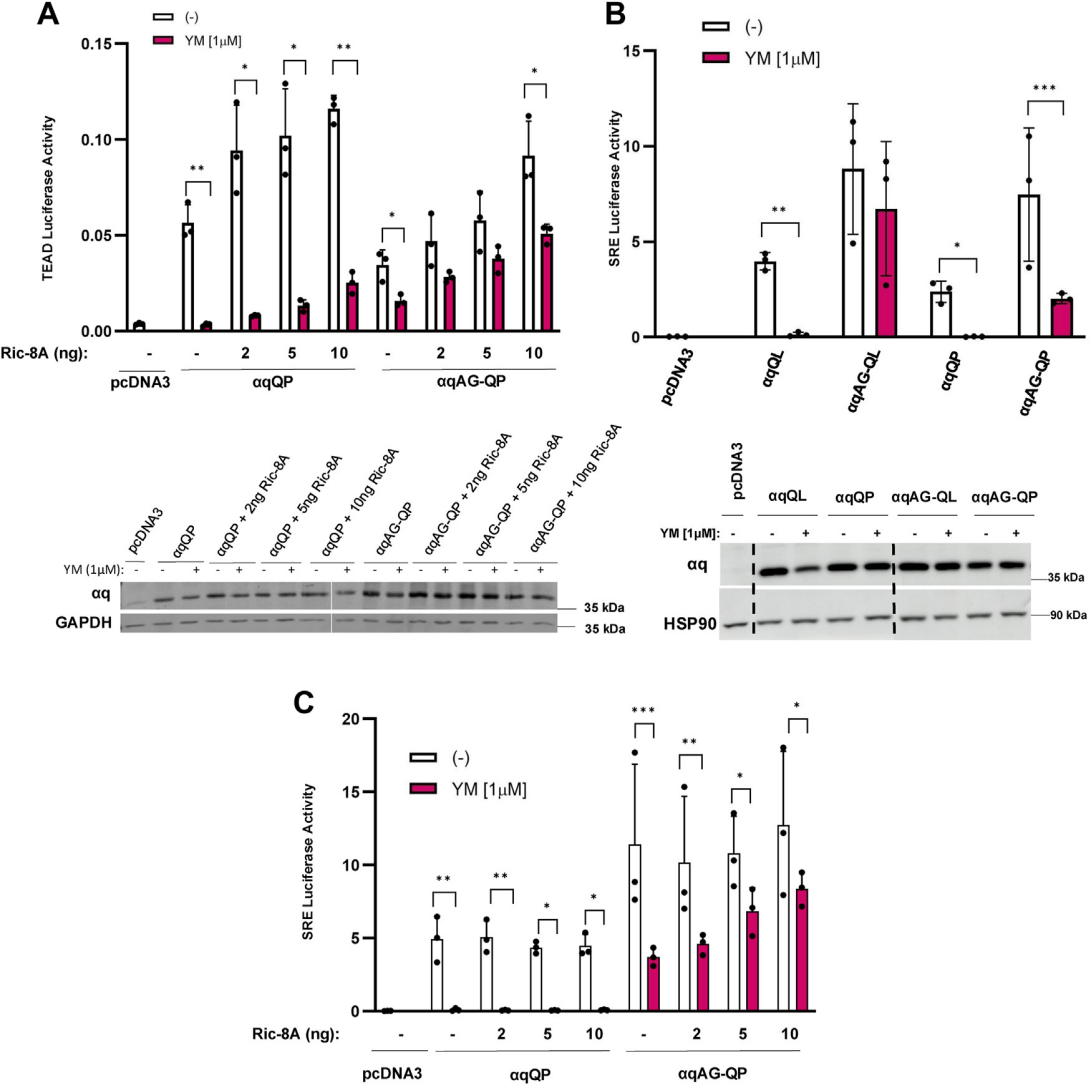
**αqAG-QP YM sensitivity is directly altered by Ric-8A.***A*, TEAD luciferase assay in *RIC-8A* KO cells transfected with either pcDNA3 (negative control), αqQP, αqAG-QP, and increasing amounts of *RIC-8A* as indicated. TEAD luciferase 8X-GTIIC reporter and renilla luciferase were cotransfected. Two hours after transfection, media were changed to serum-free and 1 μM YM was added overnight where indicated. Cells were lysed the following day and luciferase experiments were performed. TEAD luciferase values were normalized to renilla luciferase values for the same well and averaged for each condition. *B* and *C*, SRE-luciferase reporter assays in q/11 KO cells transfected with pcDNA3 (negative control), αqQL/P, or αqAG-QL/P, and SRE and renilla luciferase plasmids. Where indicated, increasing amounts of *RIC-8A* were added*.* The same protocol was followed as in *A.* Lysates were immunoblotted to assess αq protein levels (*bottom*, *B*). SRE values are normalized to their respective renilla value and averaged for each condition. Results are shown as mean ± SD and statistical significance is indicated. (n = 3, ∗*p* < 0.05; ∗∗*p* < 0.01; ∗∗∗*p* < 0.005, two-way ANOVA, Šidák's multiple comparison’s test). SRE, serum response element.

To assess if Ric-8A also affects the YM-sensitivity of another widely reported uveal melanoma CA mutant of αq, we repeated the experiment shown in Figure 3*A*, but this time using αqQP and αqAG-QP. We found that similar to αqAG-QL, αqAG-QP gained sensitivity to YM inhibition in the TEAD luciferase assay when Ric-8A was absent, but regained resistance to YM when low levels of exogenous Ric-8A were present (Fig. 5*A*). YM completely inhibited αqQP signaling and showed a slight loss of inhibition with increasing expression of Ric-8A (Fig. 5*A*), but this gain of resistance to YM was not as pronounced as observed for αqQL (Figs. 3*A* and 4*A*). Although signaling by both αqAG-QP and αqAG-QL was strongly resistant to inhibition by YM when assayed by TEAD luciferase in cells with endogenous Ric-8A (Fig. 1*B*), we observed a notable difference using the SRE luciferase reporter assay. αqAG-QP, in contrast to αqAG-QL, was substantially inhibited upon cell treatment with YM in the SRE luciferase assay (Fig. 5*B*). Differences in αqQL *versus* αqQP have been described previously; although both mutants show similar signaling, αqQP, compared to αqQL, only weakly interacts with effectors and Gβγ (39, 40). In addition, a recent report suggested that QP and QL mutants of Gα subunits reside in different conformations (42). We took advantage of this observed difference in YM sensitivity of the CA αqAG mutants (Fig. 5*B*) in the SRE luciferase assay to show that increased expression of Ric-8A, in addition to endogenous Ric-8A levels, allowed αqAG-QP to become strongly resistant to YM (Fig. 5*C*). Thus, these results revealed differences in the ability of Ric-8A to regulate sensitivity to YM inhibition of αqQP and αqQL.

### **α**qAG-QL remains in an active state in the presence of YM, and Ric-8A can enhance activation of **α**qAG-QL and to a lesser extent, **α**qQL

**Figure 6:**
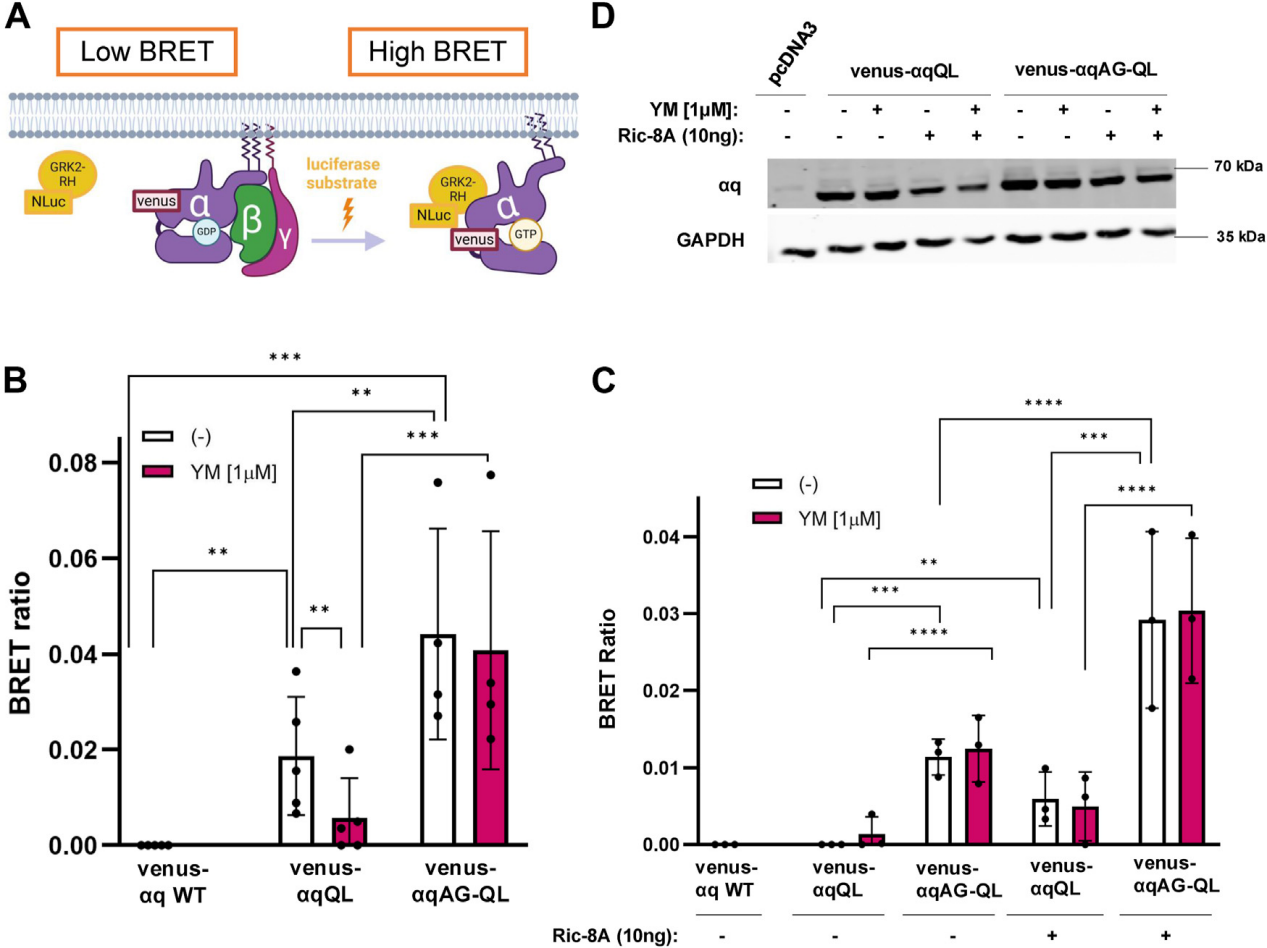
**BRET studies reveal that αqAG-QL remains GTP-bound and active in the presence of YM, and Ric-8A can increase the amount of activated αqAG-QL and αqQL.***A*, schematic depicting bioluminescence resonance energy transfer (BRET) sensors for activated GTP-bound venus-αq interaction with NLuc-GRK2-RH, which would lead to high BRET signal upon addition of luciferase substrate. *B* and *C*, HEK293 (*B*) or HEK293 *RIC-8A* KO cells (*C*) were transfected with 25 ng of Nano-luciferase fused GRK2-RH (donor) and 500 ng of Venus-fused αq (acceptor). 300 ng of Gβ and 100 ng of Gγ were coexpressed. Additionally, *RIC-8A* KO cells were transfected with 10 ng of *RIC-8A* where indicated (*C*). The following day, respective samples were treated with 1 μM YM for 2 h and BRET measurements were read 2 min after addition of Nano-glo (Promega). Venus-αq WT values were subtracted as background from all emission ratios (acceptor/donor). Results are shown as mean ± SD and statistical significance indicated (n = 4 in HEK293 and n = 3 in HEK293 *RIC-8A* KO cells, ∗∗*p* < 0.01; ∗∗∗*p* < 0.005; ∗∗∗∗*p* < 0.0001, two-way ANOVA, Šidák's multiple comparison’s test). *D*, validation of expression of venus-αq constructs in *RIC-8A* KO cells with and without Ric-8A and YM. RH, RGS-homology.

It remains unknown if Ric-8A exerts GEF activity toward αq in cells. Additionally, it is unknown in cells if αqAG-QL remains GTP-bound in the presence of YM, causing the observed resistance. To better answer these questions, we used a bioluminescence resonance energy transfer (BRET) system to further characterize αqQL and YM-resistant αqAG-QL, and to examine the effects of both YM and Ric-8A on the active, presumably GTP-bound state of these proteins. In this system, GRK2-RH-NLuc, a nano-luciferase-tagged RGS-homology (RH) domain of GRK2, is well-characterized to interact with activated, GTP-bound αq but not the inactive, GDP-bound form of αq. Thus, an increased BRET signal is expected when the donor GRK2-RH-NLuc interacts with active αq, such as αqQL, that is tagged with the BRET acceptor venus (Fig. 6*A*) (43). First, GRK2-RH-NLuc was coexpressed with venus-αq WT, venus-αqQL, or venus-αqAG-QL in HEK293 cells that have endogenous Ric-8A, and BRET ratios were generated by dividing acceptor values by donor values, and then subtracting the background venus-αq WT signal (Fig. 6*B*). We found that the BRET ratio for venus-αqQL was significantly reduced by YM, indicative of a shift to a GDP-bound, inactive conformation, whereas the BRET signal remained high for venus-αqAG-QL even with YM, suggesting it remains active and in complex with GRK2-RH. These results, paired with our data showing that αqAG-QL retains the ability to signal in the presence of YM, suggest that αqAG-QL remains in an active state and in complex with effectors more efficiently than αqQL, which cannot signal or remain bound to GRK2-RH upon YM treatment. Next, to assess the role of Ric-8A on the GRK2-RH interaction with both αq mutants, we compared *RIC-8A* KO cells ± transfected Ric-8A (Fig. 6*C*). Interestingly, though venus-αqQL was expressed sufficiently, it did not bind GRK2-RH in the absence of Ric-8A, while venus-αqAG-QL interacted with GRK2-RH with and without YM. With addition of Ric-8A, venus-αqQL interacted with GRK2-RH, even in the presence of YM. This could be due to a Ric-8A-dependent shift of venus-αqQL to the GTP-bound conformation, which supports results seen in our signaling assays where YM-sensitivity of αqQL was decreased when small amounts of exogenous Ric-8A were added back (Figs. 3*A* and 4*A*). Therefore, it was not entirely surprising to see that YM did not have much impact on the BRET ratio when Ric-8A was overexpressed. The BRET ratio for venus-αqAG-QL was also increased basally with added Ric-8A, while still maintaining resistance to YM, and was much higher than the BRET ratio for venus-αqQL, suggesting a greater proportion of venus-αqAG-QL remained in an activated state (Fig. 6*C*). Importantly, in *RIC-8A* KO cells, the increased BRET signal for venus-αqQL and venus-αqAG-QL when Ric-8A was coexpressed (Fig. 6*C*) was not a result of higher expression of the αq mutants when Ric-8A was coexpressed compared to expression in the absence of Ric-8A. In fact, under the conditions of the BRET assay, there was a slight decrease in venus-αqQL and venus-αqAG-QL expression when Ric-8A was coexpressed (Fig. 6*D*). The higher BRET signal for venus-αqAG-QL compared to venus-αqQL and the visible impact of Ric-8A on both CA αq proteins suggest that increasing the amount of GTP-bound CA αq, potentially enhanced by membrane association, is important for the observed YM-resistance.

**Figure 7:**
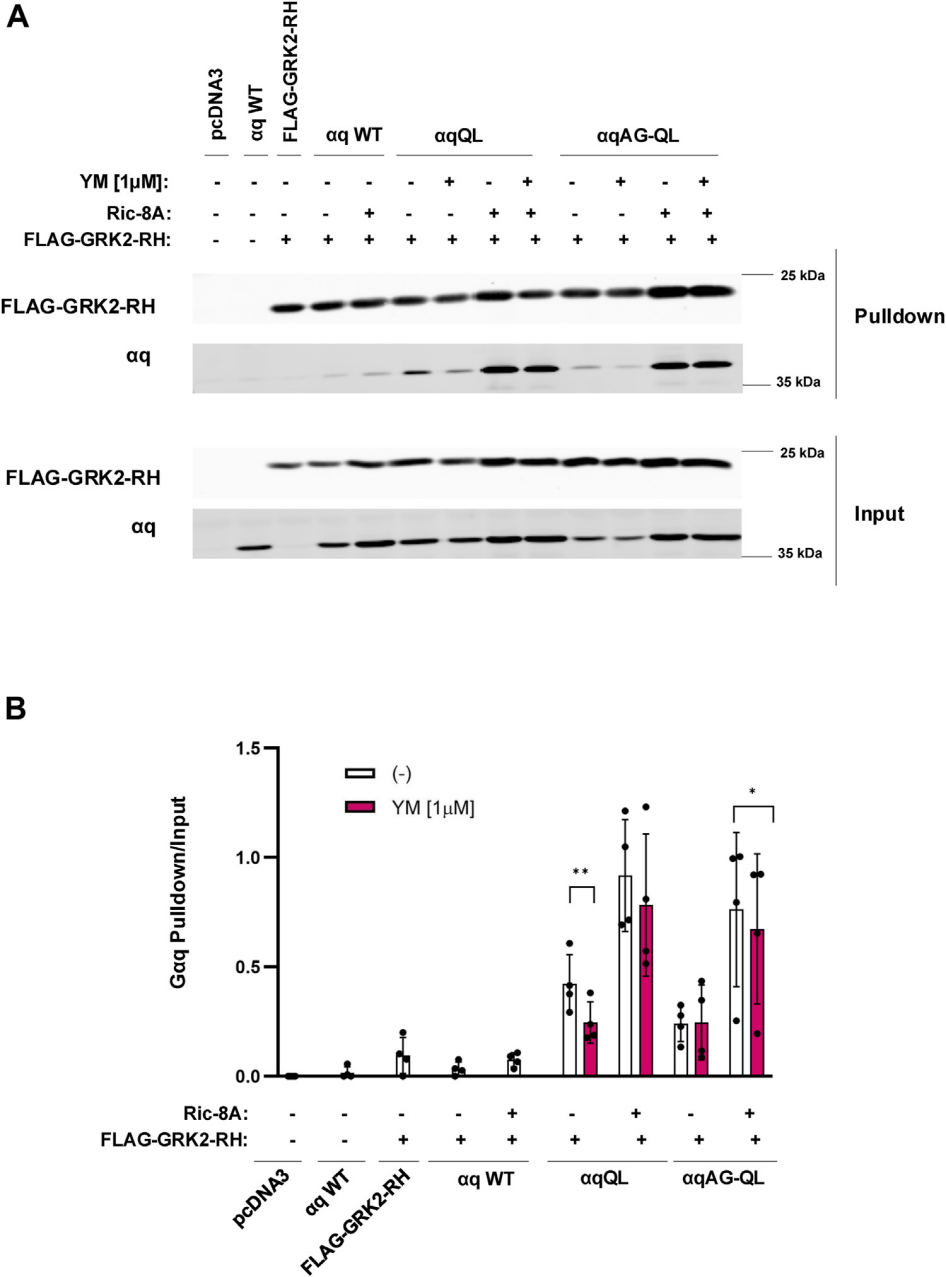
**αqQL and αqAG-QL bind GRK2-RH more strongly in the presence of Ric-8A.** A and *B*, cells were transfected with FLAG-GRK2-RH and either αqQL or αqAG-QL with and without 10 ng of *RIC-8A*. As negative controls, *RIC-8A* KO cells were transfected with pcDNA3, αq WT, FLAG-GRK2-RH, αq WT + FLAG-GRK2-RH, or αq WT + FLAG-GRK2-RH + 10 ng of *RIC-8A.* Three hours after transfection, 1 μM YM was added where indicated overnight. *A*, cells were lysed the day after transfection, and FLAG beads were used to immunoprecipitate FLAG-GRK2-RH, and the pulldowns and inputs subsequently immunoblotted for the proteins indicated. *B*, the αq pull-down signal intensities were quantified and normalized to their respective input signal intensity. Results are shown as mean ± SD. Statistical significance is indicated (n = 4, ∗*p* < 0.05; ∗∗*p* < 0.01, two-way ANOVA, Šidák's multiple comparison’s test). RH, RGS-homology.

To complement our BRET assays, we used *RIC-8A* KO cells to perform FLAG-GRK2-RH coimmunoprecipitations of the αq variants (Fig. 7). As a control, αq WT did not coimmunoprecipitate with GRK2-RH but did so very weakly with added Ric-8A, suggesting that αq GTP-bound state was necessary for interaction with GRK2-RH (Fig. 7*A*). αqQL and αqAG-QL both coimmunoprecipitated, albeit weakly, with FLAG-GRK2-RH in the absence of Ric-8A. Notably, YM significantly decreased the amount of αqQL, but not αqAG-QL, coimmunoprecipitated with FLAG-GRK2-RH (Fig. 7, *A* and *B*). The addition of exogenous RIC-8A was sufficient to abolish the effects of YM on the interaction of αqQL with FLAG-GRK2-RH, as evidenced by a strong interaction between αqQL and FLAG-GRK2-RH that was maintained in the presence of YM. In the absence of Ric-8A, treatment with YM caused a 42% decrease in the amount of αqQL pulled down with FLAG-GRK2-RH; however, upon expression of Ric-8A, similar levels of αqQL were pulled down with FLAG-GRK2-RH ± YM (Fig. 7, *A* and *B*). Of note, both αqQL and αqAG-QL experience a basal increase in interaction with GRK2-RH upon addition of Ric-8A, which could be due to increased production of functional αq as a whole, but because this increase in the coimmunoprecipitation was more dramatic than in the input sample, Ric-8A may have also increased the fraction of CA αq in the activated, GTP-bound conformation that became available to interact with GRK2-RH (Fig. 7*A*). Overall, these studies demonstrate a direct role for Ric-8A in modulating the efficacy of YM inhibition of CA αq in various contexts including signaling, in cell BRET assays, and pull-down studies.

## Discussion

The depsipeptide YM binds to and inhibits αq by locking it in an inactive, GDP-bound state. YM also effectively inhibits signaling by CA mutants of αq, such as αqQL. This has presented a paradoxical challenge to understand how YM can inhibit αqQL, which is assumed to be in an active, GTP-bound form (27, 28, 29, 30, 31, 32). Our previous work showed that αqQL redistributed off of the membrane and that signaling was strongly inhibited when cells were treated with YM, while αqAG-QL remained PM-localized and capable of robust signaling in the presence of YM (34). This previous work suggested one mechanism of how YM inhibits αqQL is to promote αqQL redistribution to a cytosolic location where it cannot engage membrane-bound effectors. However, it remains in question why enhanced membrane localization confers YM-resistance to αqAG-QL. Here, we provide further insight into this question by identifying Ric-8A as a protein that can regulate the sensitivity of CA αq mutants to YM inhibition of signaling. This work is the first to highlight that a Gαq modulatory protein impacts the ability of YM to exert its inhibitory effects on CA αq mutants.

Ric-8A is well-characterized as a chaperone protein for newly synthesized Gα subunits of the αq, αi, and α12/13 families. Ric-8A binds to and assists in protein folding of nascent Gα in a nucleotide-free state, and is then thought to catalyze the first GTP-binding event of Gα, which triggers a release of nascent Gα-GTP from Ric-8A (35, 44, 45). Mechanistically, the binding of Ric-8A to Gα has similarities to GPCRs and other non-GPCR GEFs, and Ric-8A can exert GEF activity on purified, GDP-bound Gα. However, it remains unclear if Ric-8A functions in cells as a GEF to activate signaling by Gα or if the GEF activity of Ric-8A is confined to its role as a chaperone. In this regard, some studies have postulated a role for Ric-8A in activating Gα signaling. Studies in *Caenorhabditis elegans* have shown a role for Ric-8 in αq-regulated synaptic transmission (46), and Ric-8 has been demonstrated to be important for αi/o-regulated mitotic spindle positioning in *C. elegans*, *Drosophila*, and mammalian cells (47, 48, 49, 50, 51, 52, 53). Another study showed that activation of αq by treatment with the GPCR agonist carbachol promoted PM translocation of Ric-8, suggesting a signaling role for Ric-8 that coordinates with GPCR activation of αq (54). Furthermore, previous studies reported that an engineered, myristoylated Ric-8A in HEK293 cells enhanced calcium signaling by promoting α15 activation and increased ERK activation by a Gq-coupled receptor, providing additional evidence that Ric-8A could play a signaling role in activating membrane-bound subunits of the αq family (55, 56); what is particularly intriguing is that it could mirror the dramatic effects that Ric-8A exerted on our myristoylated αqAG-QL. In addition to our existing model of the importance of αq membrane dissociation for YM efficacy, here, our results suggest that αqAG-QL may exist in an activated, GTP-bound state that escapes YM-inhibition due to the activating effects of Ric-8A. Moreover, αqQL is also susceptible to these activating effects by Ric-8A, albeit to a lesser extent.

Our results show that the loss of Ric-8A directly impacts the YM sensitivity of both αqQL and αqAG-QL. In *RIC-8A* KO HEK293 cells, we discovered an unexpected gain of YM sensitivity by the typically YM-resistant αqAG-QL that was abolished with reexpression of Ric-8A. Importantly, we also noted a gain of YM resistance with increasing *RIC-8A* concentrations with αqQL, which is potently inhibited by YM in the presence of endogenous Ric-8A. αqQL signaling in the TEAD luciferase assay was only inhibited by ∼60% in the presence of overexpressed Ric-8A and YM, compared to >95% inhibition by YM in the absence of Ric-8A (Fig. 3*A*). Furthermore, supporting this observation are the experiments showing a greater potency for YM inhibition of expressed αqQL in *RIC-8A* KO cells compared to HEK293 αq/11 KO cells (Fig. 3*C*). When Ric-8A was not expressed, the YM sensitivity of αqQL was increased, with an IC_50_ of 0.8 nM compared to 11.2 nM in cells containing endogenous Ric-8A. Because αqQL expresses and signals in the absence of Ric-8A (Fig. 3*A*), this increased YM sensitivity suggests that Ric-8A may stabilize and promote an activated form of αqQL that is more difficult for YM to inhibit. In diseases driven by CA αq mutants, such as uveal melanoma, we can speculate that inhibition of Ric-8A function could allow for more effective inhibition of mutant αq by YM (57).

To characterize the activation state of αqAG-QL compared to αqQL in cells that have endogenous Ric-8A, our BRET studies support our hypothesis that αqAG-QL remains in a more active form in the presence of YM compared to αqQL. We found that with addition of YM, αqAG-QL, but not αqQL, remains in complex with GRK2-RH, which only binds GTP-bound, active Gα (Fig. 6*B*) (43). This could explain why αqAG-QL maintains signaling capabilities and is refractory to the effects of YM. To assess how Ric-8A impacts the activation state of these proteins, we performed BRET in *RIC-8A* KO cells (Fig. 6*C*) and found further evidence for a role for Ric-8A in YM-resistance and in facilitating interactions with effectors by promoting an activated state. Surprisingly, αqQL interaction with GRK2-RH is too weak to be detectable, but in the presence of overexpressed Ric-8A, a measurable interaction is evident and YM fails to alter this interaction. These findings complement our signaling assays in which αqQL gains YM-resistance when Ric-8A is overexpressed (Figs. 3*A* and 4*A*). αqAG-QL has a detectable interaction with GRK2-RH that is refractory to YM-inhibition in the absence of Ric-8A. When Ric-8A is overexpressed, we observed an increase in BRET between GRK2-RH and αqAG-QL that was also not inhibited by YM (Fig. 6*C*). The increase in interactions between GRK2-RH and αqQL or αqAG-QL in Ric-8A KO cells upon addition of *RIC-8A* are not simply due to major changes in αq expression (Fig. 6*D*). Our GRK2-RH pull-down experiments in *RIC-8A* KO cells in Figure 7 complement our BRET assays in that for both αqQL and αqAG-QL, Ric-8A enhances interaction with GRK2-RH and the YM-sensitivity demonstrated by αqQL was abolished when *RIC-8A* was reintroduced. Notably, the change in the amount of αq pulled down with GRK2-RH when Ric-8A was expressed was drastic compared to the minimal change in expression of αq in the input ± Ric-8A. Together, these GRK2-RH interaction results show that Ric-8A, possibly through its GEF function, is able to promote a greater proportion of αqQL and αqAG-QL in an active state.

The demonstrated YM sensitivity of αqAG-QL in signaling assays (Fig. 3*A*) and the decrease in effector binding in *RIC-8A* KO cells (Figs. 6*C* and 7) suggests that without Ric-8A, αqAG-QL exists in a less stable, less active conformation that can be inhibited by YM more easily. One potential explanation is that there are both GDP-bound and GTP-bound pools of αqQL and αqAG-QL in the cell. A recent study radiolabeled cells with phosphate and assessed the nucleotide-binding state of immunoprecipitated αqQL and αq WT. The authors found that only GDP was detected bound to αq WT whereas only a small amount of GTP and a substantial amount of GDP were detected bound to αqQL (27). With a majority of αqQL being bound to GDP, this raises an important question of whether these CA mutants need to be GTP-bound to be active. Alternatively, this small pool of GTP-bound αqQL may be highly active and the main contributor to constitutive activity, and a GEF like Ric-8A may be able to directly impact the amount of GTP-bound αq. This could also explain why a typically YM-resistant αqAG-QL is sensitive to YM in a Ric-8A-free environment, where perhaps the pool of GTP-bound αqAG-QL is slightly smaller than under endogenous Ric-8A conditions. A related explanation proposes that Ric-8A can affect the different conformations in which a CA αq exists. A recent study used NMR and molecular dynamics simulations to demonstrate that catalytic glutamine mutants of αi1 could assume multiple conformations. It was found that the QL mutant of αi1 existed in additional conformations compared to GTP-bound WT αi1. Moreover, QL, QR, and QP activated mutants of αi1 reside in multiple distinct conformations (42). If there are potentially multiple activated states that mutant αq proteins exist in, it is reasonable to suggest that Ric-8A may stabilize certain favorable active conformations through its GEF and/or chaperone functions.

The ability of Ric-8A expression or enhanced membrane targeting of αq to affect sensitivity to YM is specific to CA αq and not αq WT. As described, signaling by expressed αqQL in *RIC-8A* KO cells was completely inhibited by YM, but reexpression of Ric-8A resulted in only partial inhibition by YM (Figs. 3*A* and 4*A*). On the other hand, YM maintained >95% inhibition of signaling by GPCR-stimulated αq WT regardless of reexpression of increasing amounts of *RIC-8A* (Fig. 4*B*). Likewise, the added myristoylation site alone was insufficient to confer YM resistance to αq WT. Our previous work revealed that αqAG WT is fully sensitive to YM as signaling to SRE/TEAD reporters and production of pERK by carbachol-stimulated αqAG WT was completely inhibited by YM, identical to αq WT (34). These results highlight clear differences in the regulation of YM inhibition of αq WT compared to αqQL inside the cell. αq WT should exist in an inactive, GDP-bound form and therefore be readily available to bind YM, but αqQL likely has more reliance on cellular mechanisms to drive it into an inactive form that is able to be inhibited by YM.

Additionally, our results show that YM-resistance due to enhanced membrane binding is not restricted to αqQL; αqQP (Fig. 1) and a YM-sensitive α13QL (Fig. 2) both lose sensitivity to YM upon addition of an N-terminal myristoylation site. αqQP is biochemically different from αqQL, though their signaling capabilities are comparable (Fig. 1) (39, 40). αqQP was shown to bind much more weakly to many effectors compared to αqQL in BRET and pull-down experiments. Authors attributed this to a difference in conformation of αqQP compared to αq WT or αqQL in the Switch II region that could be a result of the bulkier proline residue compared to the leucine in αqQL (40). Interestingly, in our studies using cells with endogenous Ric-8A, introduction of myristoylation in αqAG-QP conferred YM-resistance similarly to αqAG-QL *via* TEAD luciferase reporter assay measurements (Fig. 1*B*). Our studies with α13QL, belonging to an unrelated G protein family, mutated to contain key YM-binding residues (41) demonstrated that enhanced membrane localization due to introduction of a myristoylation site was likely the cause of YM-resistance, not some other features within the backbone of αq (Fig. 2). Efforts in engineering YM binding sites into αi, α16, and αs have been successful and provide valuable tools for further elucidating both the YM mechanism as well as Gα cellular regulation (29, 58, 59). Here, with CA α13, we confirm that myristoylation overcomes any YM inhibitory effects, highlighting the importance of membrane binding as a critical component that regulates YM inhibition of CA Gα.

Our results with αqAG-QP further highlight the ability of Ric-8A to regulate the sensitivity to YM. Like αqAG-QL in *RIC-8A* KO cells, Ric-8A had a similar effect on αqAG-QP of lost resistance to YM, but resistance was restored by reintroduction of *RIC-8A* (Fig. 5*A*). However, to our surprise, SRE reporter assay measurements in cells containing endogenous Ric-8A (Fig. 5*B*) showed that αqAG-QP demonstrated detectable sensitivity to YM. Similarly, αqAG-QP displayed partial sensitivity to YM in the pERK readout (Fig. 1, *C* and *D*). To take advantage of this unique vulnerability of αqAG-QP in the SRE reporter assay, we found that increasing amounts of *RIC-8A* recovered YM-resistance exclusively for the myristoylated mutant, while αqQP remained unaffected by overexpressed Ric-8A (Fig. 5*B*). This suggests that αqAG-QP may exist in a less favorable active conformation, potentially similar to αq WT, as previously suggested (42), and that Ric-8A can help to shift it into a more active conformation that is refractory to the effects of YM.

Other proteins likely contribute to regulating the sensitivity of αqQL to YM and the differences in YM-sensitivity of αqAG-QL compared to αqQL. If CA Gα are more susceptible to GEF activity than previously appreciated, then one advantage that αqAG-QL may possess over αqQL could be related to its enhanced proximity to GPCRs or other non-GPCR GEFs due to its sustained membrane localization. In this scenario, αqAG-QL, compared to αqQL, would be more readily activated and maintained in a YM-resistant form due to its enhanced membrane localization. Recent studies have highlighted that CA αq may not be entirely unaffected by GPCRs. Interestingly, one study found that αqQL/P activation could be attenuated by adding a neurotensin receptor allosteric modulator, suggesting receptors can in some cases enhance signaling (42). Another group showed that in response to muscarinic acetylcholine receptor (mAchR) activation, signaling from αqQL-expressing cells increased and subsequently decreased in the presence of muscarinic acetylcholine receptor antagonist atropine (27). Another interesting study utilized FRET experiments to show that αqQL and αq WT bound to activated m3AchR equivalently (60). Thus, GPCR-mediated GDP/GTP exchange may contribute to the activation of CA αq and likewise play a role in maintaining αqAG-QL in a YM-resistant state. In addition to the potential for increased interaction with GPCRs or other GEFs, αqAG-QL, compared to αqQL, may also remain in close proximity to its effectors, even in the presence of YM. Lastly, it will be important to consider how interaction of Gβγ with CA αq mutants affects sensitivity to YM. A recent structural study indicated that in addition to functioning as a guanine nucleotide dissociation inhibitor, YM also makes contact with Gβγ subunits in the context of a Gq heterotrimer, thereby promoting heterotrimer formation as an additional mechanism for locking αq in an inhibited state (61). Interaction with Gβγ is critical for signaling of αqQL and αqQP (39), not just αq WT, and Gβγ may play a critical role in regulating the sensitivity to YM of CA αq mutants.

Together our data point toward a novel and direct effect of Ric-8A on the ability of YM to inhibit CA αq, likely achieved by shifting inactive αqQL to more stable and activated GTP-bound conformations. Therefore, Ric-8A should be considered more seriously in future studies as a signal enhancer of αqQL. These and other studies also raise the possibility that CA αq may be susceptible to GEF activity and therefore GDP-bound CA αq species may be more prevalent in cells than previously appreciated. A better understanding of the regulation of these oncogenic CA αq proteins could lead to more informed approaches toward inhibiting them.

## Experimental procedures

### Plasmids and antibodies

Hemagglutinin-tagged αq WT, hemagglutinin-tagged αqQL/P, and αqAG-QL in pcDNA3 were described previously (39, 62, 63, 64). αqAG-QP in pcDNA3 was made using site-directed mutagenesis from αqAG WT (63) using the primers (Forward: 5′-cgatgtagggggcccaaggtcagagagaag– 3′, Reverse: 5′ -cttctctctgaccttgggccccctacatcg–3′). FLAG-tagged α13QL and α13QL-YS were described previously (41). α13AG-QL and α13AG-QL-YS were made using site directed mutagenesis using the primers (Forward: 5′-cggatccgccaccatgggggccggcgcgtcgtcgcggtccgtgctg-3′, Reverse: 5′-cagcacggaccgcgacgacgcgccggcccccatggtggcggatccg-3′). myc-β1 and γ2 expression vectors have been previously described (65). Ric-8A in pcDNA3.1 has also been previously described (66). BRET constructs Venus-tagged αq WT and Venus-tagged αqQL and Nanoluciferase-tagged GRK2-RH in pcDNA3.1 (40, 67) were generously provided by Mikel Garcia-Marcos (Boston University). Site-directed mutagenesis was used to design Venus-tagged αqAG-QL using the primers (Forward: 5′-agcgaggcacttcggaagaatcgatctggagtgcatcatgggatgctgcctgag-3′, Reverse: 5′-ctcaggcagcatcccatgatgcactccagatcgattcttccgaagtgcctcgct-3′). FLAG-tagged GRK2-RH was generated by synthesizing DNA (GenScript) coding for amino acids 45 to 178 of GRK2 plus a C-terminal linker (GGGGSGGGGS) and FLAG tag (DYKDDDDK), followed by subcloning into pcDNA3.

For Western blotting, the following primary antibodies were used. The pERK1/2 (Cat. #9101S) and ERK1/2 (Cat. #4696S) antibodies were from Cell Signaling Technologies. αq (Cat. #13927-1-AP), GAPDH (Cat. #60004-1-Ig), and Ric-8A (Cat. #66625-1-Ig) were from Proteintech. FLAG (Cat. #F1804) for detection of α13 and GRK2-RH in the immunoprecipitations was from Sigma-Aldrich. HSP90 (Cat. #sc-7947) was from Santa Cruz Biotechnology. The secondary antibodies IRDye 680RD goat anti-rabbit IgG (H+L) (Cat. #92568071) and IRDye 800CW donkey anti-mouse IgG (H+L) (Cat. #92532212) used to visualize proteins on immunoblots were purchased from LI-COR.

### Cell culture and reagents

HEK293 q/11 KO cells (30) were generously provided by Dr Asuka Inoue. HEK293 *RIC-8A* KO cells were described previously (66). HEK293 cells, received from American Type Culture Collection were cultured in minimal essential media (Cat. #10–010-CV), and HEK 293 q/11 and *RIC-8A* KO cells were cultured in Dulbecco's modified Eagle's medium (Cat. #10–017-CV) from Corning). All media were supplemented with 10% fetal bovine serum (Cat. #900–108 from Gemini) and 1% penicillin/streptomycin (Cat. #P4333 from Sigma-Aldrich). Lipofectamine 2000 (Cat. #11668–019) from Invitrogen or Pei MAX (Cat. #24765–100) from Polysciences were used for all transfections. YM-254890 (YM) (Cat. #257–00631) was from Wako Chemicals USA Inc. Carbachol was from Sigma-Aldrich (Cat. #C4382). Cell culture plates and materials were from GenClone, Costar, or Fisher.

### Dual luciferase assay

HEK293 q/11 KO, HEK293 *RIC-8A* KO, or HEK293 cells were cultured in 12 well plates and cotransfected with a Gα construct, the Renilla luciferase control plasmid, and either the TEAD luciferase 8X-GTIIC reporter (gifted from Stefano Piccolo, Cat. #34615, Addgene) or pSRE-luciferase reporter (68). In some cases where specified, *RIC-8A* plasmid was also transfected. For αq WT experiments, m3AChR was cotransfected (Fig. 4*B*). Lipofectamine or PEI Max was used according to manufacturer’s instructions and the media were changed to serum-free media with or without YM (concentrations specified in figure legend) 2 h after transfection. In some cases where agonist-stimulated αq signaling was measured, 100 μM carbachol was added 1 h after YM treatment. Sixteen hours later, cells were lysed in 1X passive lysis buffer provided by Promega dual luciferase assay kit (Cat. #1960) and plated in a white opaque 96-well plate in triplicate. Luciferase activity was detected by the GloMax Explorer luminometer. To run Western blots on these samples, 20 μl of the lysate was diluted in 5x SDS sample buffer and ran on 10% SDS-PAGE gels as described below following our Western blotting protocol.

### GRK2-RH immunoprecipitations

Immunoprecipitations were performed similarly to other reports (34, 40). HEK293 *RIC-8A* KO cells were transiently transfected with FLAG-tagged GRK2-RH and αq WT, αqQL, αqAG-QL with or without Ric-8A. pcDNA3, αq WT alone, and GRK-2RH alone were transfected alone as controls. Three hours after transfection, media were changed and 1 μM YM was added to respective plates overnight. The next day cells were washed with PBS and lysed in 350 μl of lysis buffer C (20 mM Hepes pH 7.5, 100 mM NaCl, 0.7% Triton X-100, 5 mM MgCl_2_, and 1 mM EDTA supplemented with 1 mM DTT, 1 mM PMSF, 2 μg/ml leupeptin, and 2 μg/ml aprotinin, and 0.1 × cOmplete mini protease inhibitor cocktail). Lysates were incubated on ice for 30 min and centrifuged for 10 min at 13,000 rpm. Briefly, 50 μl of the lysate was saved for input while the rest of the supernatant was saved for the immunoprecipitation. In addition, 40 μl of ANTI-FLAG M2 Affinity Gel bead slurry (Cat. #A2220 Sigma-Aldrich) per sample was aliquoted to new tubes and washed 3x with lysis buffer C. The remaining supernatant was added to the bead slurry and rotated end-over-end for 2 h at 4 °C. The beads were then pelleted by centrifugation for 1 min, supernatant was removed, and then the beads were washed 3x with lysis buffer C. Protein was eluted from the beads in 80 μl of 1X SDS sample buffer with 1% β-mercaptoethanol. Input and pull-down samples were run on 15% SDS-PAGE gels, transferred onto a nitrocellulose membrane, and probed with antibodies for αq and FLAG to detect GRK2-RH (antibodies referenced above). Bands were visualized using LI-COR secondary antibodies, followed by imaging on LI-COR Odyssey F imager, and bands were quantified on ImageJ (https://imagej.nih.gov/ij/), normalizing each pulldown to their respective lysate.

### pERK/ERK immunoblotting

HEK293 q/11 KO cells were transfected with αq WT, αqQL, αqAG-QL, αqQP, or αqAG-QP. The next day, cells were changed to serum-free media, and respective samples treated with 1 μM YM overnight. Cells were lysed the following day in 1X SDS sample buffer with 1% β-mercaptoethanol and run on 10% SDS-PAGE gels, transferred to a nitrocellulose membrane, and probed with antibodies referenced above for pERK, ERK, αq, and GAPDH. LI-COR secondary antibodies referenced above were used to visualize the protein, and blots were imaged on the LI-COR Odyssey F imager. Bands were quantified as phosphorylated ERK divided by total ERK on ImageJ (https://imagej.nih.gov/ij/).

### Western blotting

Protein lysates were run on 10% SDS-PAGE gels for all immunoblots with the exception of GRK2-RH immunoprecipitations, which were run on 15% SDS-PAGE gels. Lysates were transferred to nitrocellulose membranes (Cat. #nc9680617) and subsequently blocked in either 2.5% bovine serum albumin (for pERK blots) or 5% milk (all other blots) in 1x Tris-buffered saline (TBS)/0.05% Tween 20 at room temperature for 1 h. The blots were incubated with their corresponding primary antibodies in 2.5% bovine serum albumin or 5% milk in 1x TBS/0.05% Tween 20 at 4 °C overnight. The blots were then washed 3x in 1x TBS/0.05% Tween 20 at room temperature and then incubated with the LI-COR antibodies referenced above for 1 h at room temperature. Blots were again washed 3 times in 1x TBS/0.05% Tween 20 and once with PBS before imaging on the LI-COR Odyssey F imager.

### Bioluminescence resonance energy transfer

BRET experiments were adapted from previously published methods (43, 67). Briefly, HEK293 or HEK293 *RIC-8A* KO cells were seeded in 6-well plates at 350,000 cells/well. The next day, cells were transfected with Nanoluciferase-fused GRK2-RH (donor) and αq WT, αqQL, or αqAG-QL fused to Venus (acceptor) at a 1:20 donor:acceptor ratio. Unstimulated αq WT alone was used as a negative control. Gβ and Gγ constructs were also coexpressed. In *RIC-8A* KO cells, *RIC-8A* was also transfected at the same time as the BRET constructs as indicated. The following day, designated samples were treated with 1 μM YM for 2 h. Cells were washed with PBS, and then 1 ml of fresh PBS was added to scrape and collect cells. Cells were centrifuged for 5 min at 550*g* at room temperature. The supernatant was aspirated, and cells were gently resuspended in 900 μl PBS. In the dark, 50 μl of PBS was added to designated wells of a 96-well plate (Cat. #07–000–128, Thermo Fisher Scientific) used for the assay. Subsequently, 50 μl of cells were then added to respective wells of the 96-well plate, with technical replicates ranging from 5 to 8 per condition. Two microlitres of luciferase substrate Nano-glo, diluted 1:200, (Cat. #N1130, Promega) was added to each well. The plate was read 2 minutes later in the CLARIOstar Plus plate reader (BMG Labtech) equilibrated to 37 °C. Luminescence was measured at 450 ± 20 nm and at 535 ± 20 nm. BRET ratio was calculated by dividing acceptor (535 ± 20 nm) by donor (450 ± 20 nm) emission intensities, averaged for each condition. Unstimulated Venus-αq WT values were subtracted as background from each averaged value. Expression of the Venus-αq constructs were validated by Western blotting methods (Fig. 6*D*).

### Statistics

GraphPad Prism was used for all data analysis in results. A two-way ANOVA followed by Šidák's multiple comparison test was used to determine significance. Error bars in all experiments indicate mean ± SD with significant differences indicated as ∗*p* < 0.05; ∗∗*p* < 0.01; ∗∗∗*p* < 0.005; ∗∗∗∗*p* < 0.0001.

## Data availability

All data are contained within the paper.

## Conflict of interest

The authors declare that they have no conflicts of interest with the contents of this article.
